# Dr. Arjun Srinivasan on being pathogen agnostic, following his passion, and knowing when to lead and when to follow

**DOI:** 10.1017/ash.2024.6

**Published:** 2024-02-16

**Authors:** Arjun Srinivasan

**Affiliations:** Division of Healthcare Quality Promotion CDC, Atlanta, GA, USA

Dr. Srinivasan is the Deputy Director for Program Improvement in the Division of Healthcare Quality Promotion at the Centers for Disease Control and Prevention and a recently retired Captain in the United States Public Health Service. Before joining CDC, he was an Assistant Professor of Medicine in the Infectious Diseases Division at the Johns Hopkins School of Medicine where he was the Founding Director of the Johns Hopkins Antibiotic Management Program and the Associate Hospital Epidemiologist. His primary responsibilities include oversight and coordination of efforts to eliminate healthcare-associated infections and reduce antibiotic resistance. His research and investigative areas of concentration include outbreak investigations, infection control, multi-drug resistant gram-negative pathogens, and hospital antibiotic stewardship. Dr. Srinivasan has published more than 100 articles in peer-reviewed journals on his research in healthcare epidemiology, infection control, antimicrobial use, and resistance.

## Our readers are very familiar with your background and contributions to antimicrobial stewardship, infection prevention, and HAIs. Tell us something about your professional journey that others may not know

One thing that surprises people and frankly, is surprising to me is that I never envisioned a career at the CDC or in public health. I had no idea that this was an option during my training. I think 20 years ago, the connection between public health and healthcare was not nearly as strong as it is today, and as a result, a lot of us had little exposure to it and had no idea what people in public health did. I mean, we all knew about the CDC and HICPAC and followed their recommendations, but I never imagined working at the CDC. I was trained at Hopkins, which is a CDC Epicenter. While there, I was involved with Trish Perl’s Epicenter grant and collaborated with Denise Cardo, who at that time, was a branch chief at the CDC. And we worked together on infection prevention and stewardship projects as I was starting the antibiotic stewardship program at Hopkins. She called me one day and said, “Hey, have you ever thought about a career at CDC?” And I said, “I had no idea that was a career option.” So, I came down, interviewed with her team, saw the work they did, fell in love with all of it and never looked back. That was 20 years ago. I tell people that story often, because I think it’s a good reminder that you really don’t know how your career is going to unfold. I remember thinking as a fellow that I needed to talk to people I admire and figure out exactly how they got to where they are, because I’ve got to follow that path, or I’m not going to have a good career. Instead, I ended up with a career I love, simply because I followed my passion, and was open to opportunities that presented themselves. My advice is to find something in medicine that you really enjoy and go with that. If you do that, you’ll end up with a career you really enjoy because you’re doing what you love. Remain open minded, curious, and engaged in the pursuit of new avenues. Find a passion and follow that passion. It will end up working out well.

## So, what was your passion at that point which led you to the CDC? Is that still your passion?

When I was in training, I ended up in healthcare epidemiology because I couldn’t pick a pathogen. In ID, especially at large academic centers like Hopkins, the research is very pathogen focused. I stumbled upon healthcare epidemiology as a space where you didn’t have to pick pathogens because we deal with everything out there. There was also a connected opportunity in antibiotic stewardship. We were always taught that as an ID doctor, antibiotic use is our procedure, right? I was always really fascinated by antibiotic use, and thought “This is the coolest thing ever,” because a patient can present on death’s door, and if I can figure out the problem correctly, I can cure the patient within a matter of days with antibiotics. I mean, what an amazing specialty! So, I think that those types of observations, the confluence of all pathogens, and opportunities for stewardship made it perfect for me. I have also always enjoyed policy work and have found it interesting to take care of individual patients, but also to think about how the system could be made better for all patients. That’s what we do in healthcare epidemiology. It’s basically public health within the healthcare setting. We’re thinking about the individuals, but also working hard to protect the population, and understanding the best way to do this requires skills like communication, negotiation, and compromise. I always really enjoyed talking to people about ways to change practice, how to make policies acceptable, and how best to “sell” these to people. How do we convince them that this is the right thing to do? I always really enjoyed that. Even as a medical student, I did some work on healthcare policy related to the rollout of TennCare, which I really enjoyed. Healthcare epi was exciting to me because it presented an opportunity to conduct health policy for the healthcare system. And so, it was that confluence of all the things I really enjoyed about ID: to be pathogen agnostic, to focus on antibiotic use, to work on policy and communication. To me, it felt incredible to find a combination of all these things I really enjoyed. It’s all held true, and probably even more so now than 20 years ago. Healthcare epi is so front and center in discussions about policy about protecting health, shaping healthcare and patient safety. There is now an increasing recognition of the role that healthcare epi and stewardship play in patient safety, and it’s been interesting to see that growth. I’ve loved every minute of it. I realize that it’s not for everyone. Sometimes people say to me “I can’t imagine anything worse than shepherding the toy cleaning policy through the Infection Control committee,” but I think that’s the interesting stuff. It exercises a different kind of mental muscle and stretches that part of my personality which approaches problems by asking “help me see what you see.” It has taught me so much about patience and compromise. In healthcare epi, you’re taught to really listen to the opinions of others and not dismiss them and be able to separate the critique from the critic, which is such a fundamentally important skill. Think of how much better the world would be right now if more people actually listened to people who may not agree with them.

## Several ASHE contributors have cited your gifts as a mentor and sponsor of others, especially women. Do you have a personal mentorship philosophy? Why do you think you’ve been so successful in this area?

Every position I’ve taken was influenced by the people who were involved. To fully love the work and have fun, the people are so important. I’ve benefited enormously from people who have helped me, and I’ve been given so many opportunities by generous and gracious people. For me, mentoring is, in a sense, a moral responsibility. I wouldn’t be here had it not been for the kindness of so many others. It’s my obligation to give that same gift to other people. That has been my guiding principle. Maya Angelou said that people will forget what you did but will never forget how you made them feel. To mentor and promote people, you’re paying that feeling forward. So, you’re not only putting people in opportunities where they’re going to thrive and do amazing work, but you’re going to benefit from it, and the field is going to benefit. It’s very self-serving as well, to give the credit and the spotlight away. It feels very good.

## Who are some of your mentors who’ve been pivotal to your success over the years? What made them special?

I’ve really been so lucky to have to two incredible mentors. My first was Trish Perl who was the healthcare epidemiologist at Johns Hopkins and the person who introduced me to the world of healthcare epi and infection control. She gave me so many opportunities at every point in my training, like talks and papers. She took me to my first SHEA meeting to present our research and introduced me to everyone. She was so generous and helped me build this network of contacts in the field. And then when I came to the CDC, I worked for Denise Cardo, who had recruited me here. I followed her up the ladder when she became the director of the Division of Healthcare Quality Promotion and spent the last 10 years working directly for her until she recently retired. She was an amazing mentor, teaching me how to get things done. She had the unique skill of figuring out how to operate in very complex and challenging spaces, and within the constraints of government funding and regulations. She knew how to leverage partnerships to have a bigger impact and was remarkable at knowing when to lead and when to follow. I learned so much from her, like when to be out front and center, and very visible, versus taking a step back and finding a partner who is better suited to lead an initiative who may face less objections and controversy, and for whom opportunities may better align. My mentorship and sponsorship of women has its roots with mentorship by women. I’ve only had women as bosses for my entire career!

## On a serious note, the pandemic was an extremely challenging time for you and your colleagues in public health. How did you manage to stay optimistic, and how did you continue to inspire your team in the darkest hours?

Without question, it was the hardest period of anybody’s career. Morale was at an all-time low. Everybody was working incredibly hard and there were times when people literally pulled all-nighters to get important guidance or analysis out as fast as possible. And then to see in the paper the next day that “CDC scientists got it wrong again,” or “these guys are idiots, they don’t know what they’re doing,” or “the bureaucrats are just sitting in their in their little offices, and they don’t know anything about what’s truly going on,” etc. There were also the political challenges that ensued, and things became so polarized, and what was scientific or evidence-based was replaced by what was politically expedient and popular. I wouldn’t say that I had any special tricks to shepherding people through those times. It was made even harder by the fact that we weren’t together, and everything was virtual. It was harder to check in with people, which was really important. Setting up one-on-ones with people, even if over video, to ask, “tell me how you’re doing, because you can’t be fine. I’m not fine.” Some of it was modeling your own vulnerability by saying, “Hey, I’m really frustrated by this, and I hate that we’re getting beat up in the press for doing our best with the information at hand.” Maybe many of these things were later proven wrong or unnecessary, but at the time, we don’t know which ones those were, so we had to recommend all of it. I think part of good leadership is modeling that vulnerability for people, so they feel more comfortable voicing their frustrations. But you must do that with care without giving up on the mission because everything is terrible at that moment.

## We live in a time of many intersecting crises. What public health issues and challenges keep you up at night?

What concerns me most is not anything related to pathogens or practices. It’s that there’s going to be and already is this incredible erosion of support for healthcare epi, both within the healthcare system and in the public health arena. During the pandemic, there were incredible investments in health care epi, infection control, and stewardship programs, which were writing and constantly revising COVID treatment guidelines. We received literally billions of dollars in investments from Congress to build better, stronger systems for healthcare epi and stewardship in the public health sector, and to carry that over into the healthcare system. People recognized that this was an important investment. But as with everything, the nation has a very short memory, and we’re already seeing the rapid erosion of that support. There’s no recognition of the importance of systems we’ve built, and their role in keeping people safe beyond the pandemic. And yes, maybe we can’t sustain pandemic-level funding but there isn’t a thoughtful discussion about what is the right level of funding. There is a haste to go back to pre-pandemic levels, or frankly, in a lot of places, even less. I talk to people in hospitals who report that their Infection Control budget is being cut to even less than pre-pandemic levels. We see the same thing on the public health side at all levels, federal, state, and local. Honestly, that’s my biggest concern, that we finally built a good infrastructure and now it’s all going away. If all that foundation goes away, we’re never going to be able to make the transformative gains in patient safety that we all desire.

## On a positive note, what upcoming medical and scientific advances are you most optimistic about in terms of the greatest potential impact on the health of the public?

There is a lot I don’t understand about artificial intelligence, but I think today, if anybody asks you that question, you have to say artificial intelligence, because it’s just the answer to everything! But I do think there are some intriguing examples of harnessing the power of electronic health records and combining that data with advanced analytics as the foundation for interventions. A great example is the work of Susan Huang, Shruti Gohil and colleagues to build intelligent therapeutic guidance within an electronic health record at the Hospital Corporation of America, which uses the patient’s own information to calculate that patient’s risk of having a drug resistant infection, which led to a dramatic decrease in the use of broad-spectrum antibiotics. To me, tapping into the information that’s all sitting right there is incredibly exciting. We must figure out how to get it out, analyze it in a meaningful way, and then provide a concise report back to the provider. This seems to have huge potential in stewardship and infection control. So, I’m excited to see what people can do when we’re able to get rich information out of the electronic health record. I’ve heard a saying, that health care is data rich, but information poor. I hope that’s starting to change. We’re beginning to take the initial steps here at the CDC’s National Healthcare Safety Network, of obtaining patient-level data submitted electronically for better risk-adjustment for antibiotic use ratios,^1^ and application of AI to this could be incredibly transformative. Currently, we must ask hospitals to input all that data and cannot justify the burden, but if we can access information sitting on a server, we can get anything out of there, and it opens almost limitless possibilities. We hope that it will greatly help people guide actions. I’m really looking forward to seeing what we’re able to do when we have everybody reporting antibiotic use and resistance data to NHSN, which is a requirement now starting in 2024. Soon we’ll have about 4–5000 hospitals submitting data. Ten-to-twenty years ago, this seemed impossible, and yet here we are.

## One of your superpowers is connecting with audiences. Have you always had this gift or is it something you’ve had to work on over time? Was there a particular speaker or lecturer you encountered early on that inspired you? For our readers, what are your top 3 tips for effectively connecting with audiences?

It’s both a talent and something that I’ve worked very hard to develop. For me, successful communication comes from a from a place of passion; I love what I do, and I love every opportunity to stand up in front of people or do an interview, to tell them more about what our team at the CDC is doing. That passion comes through when I’m communicating about something that I really care about. I think every great speaker is speaking about something they truly believe in. A good speaker makes that passion come through, so that the audience gets equally excited about the topic. I still spend quite a bit of time planning and practicing my presentations, focusing on timing and transitions. So, to summarize my top tips, (1) remember that you’re passionate and love what you’re doing. Yes, you may get nervous in front of a crowd, but remember, they invited you to give that talk, because you’re smart, and know the material. Let the audience see that passion. (2) Think about the transitions and how the presentation is organized, because if you’ve organized it well, it will be easier for you to deliver. (3) Practice, practice, practice.

## Our professional community is eager to know what is next for you. What do you envision yourself doing after a long and storied career in public health? What else are you interested in pursuing?

That is the million-dollar question. I love what I do, and could easily continue to do this, but there are a lot of other interesting opportunities in public health, academics, the private sector, in foundations, in regulatory and accreditation organizations. So, I’m certainly open to great opportunities as they come along, but I don’t have one in mind at this point. As I stated earlier, the important thing is to continue to keep an open mind throughout one’s career.

## Finally, what books, articles, or podcasts are you consuming, either professionally-oriented, or for leisure, that you would recommend to our readers?

There’s a lot I’ve consumed that I’ve found very meaningful. There’s a TED talk by Simon Sinek addressing the “how”, the “what” and the “why” of leadership.^2^ I think it should be required viewing for everybody. Let’s face it, nobody works in healthcare epi, stewardship, or public health for the money, right? So, there’s, a real core of “why” to what motivates us. Seeing how he frames that was really eye opening. As a leader, if you can get people focused on the “why” then they’re going to have such passion for what they’re doing and everything else falls away. In healthcare epi, in stewardship and public health we’re saving lives. The “why” is that people are at risk, and we are protecting them. I mean, how can you have a higher calling than that?

I also really enjoy Jim Collins’ book *Good to Great* about leadership.^3^ There’s also *The Undoing Project* by Michael Lewis, about the friendship between Daniel Kahneman and Amos Tversky.^4^ Michael Lewis is such a great storyteller. Another one of his books, *The Fifth Risk* should be required reading for anyone who has worked for the government or in public health.^5^ It basically talks about the fundamental importance of programs, and investment, stewardship and tending of programs, and if we don’t do that, we will be in a world of hurt. So, all of these have led me to think about things differently and have enriched my passion for public health, healthcare epi and stewardship immeasurably.
